# Psychosocial Outcomes and Quality of Life in Patients with Hemophilia a Without Inhibitors: The HemoLIFE Study

**DOI:** 10.3390/jcm15051790

**Published:** 2026-02-27

**Authors:** María Teresa Álvarez-Román, Ramiro José Núñez Vazquez, Olga Benítez Hidalgo, Laura Quintana Paris, Laura Entrena Ureña, Francisco José López Jaime, Hortensia De la Corte-Rodríguez, María García Dasí, Pau Bosch, Carmen Álvarez Cuervo, Itziar Guerra Garaeta, Virginia Barras Sanchez, Inmaculada Soto-Ortega

**Affiliations:** 1 Unidad de Trombosis y Hemostasia, Hospital Universitario la Paz, 28046 Madrid, Spain; 2 Unidad de Hematología y Hemoterapia, Hospital Universitario Virgen del Rocío, 41013 Sevilla, Spain; ramirojosenv@gmail.com; 3 Servicio de Hematología, Vall d’Hebron Barcelona Hospital Campus, 08035 Barcelona, Spain; olbehi21@gmail.com; 4 Servicio de Hematología y Hemoterapia, Hospital Universitario de Gran Canaria Doctor Negrín, 35010 Las Palmas de Gran Canaria, Spain; lauraquintanaparis@gmail.com; 5 Servicio de Hematología y Hemoterapia, Hospital Universitario Virgen de las Nieves, 18014 Granada, Spain; laura_eu@hotmail.com; 6 Servicio de Hematología y Hemoterapia, Hospital Regional Universitario de Málaga, 29010 Málaga, Spain; franlopezjaime@gmail.com; 7 Servicio de Medicina Física y Rehabilitación, Hospital Universitario La Paz, 28046 Madrid, Spain; hortensia.corterodri@salud.madrid.org; 8 Instituto de Investigación IdiPAZ, 28046 Madrid, Spain; 9 Universidad Alfonso X El Sabio, 28691 Madrid, Spain; 10 Independent Researcher, 46026 Valencia, Spain; mariagarcia1984@hotmail.com; 11 Unidad de Trombosis y Hemostasia, Hospital Universitario y Politécnico de La Fe, 46026 Valencia, Spain; pau_boschfer@hotmail.com; 12 Departamento Médico, Roche Farma S.A., 28042 Madrid, Spain; carmen.alvarez@roche.com (C.Á.C.); itziar.guerra@roche.com (I.G.G.); virginia.barras_sanchez@roche.com (V.B.S.); 13 Sección de Hemostasia y Trombosis, Hospital Universitario Central de Asturias, 33011 Oviedo, Spain; isotor60@gmail.com

**Keywords:** hemophilia A, productivity, psychological adjustment, quality of life

## Abstract

**Background:** Hemophilia adversely affects several health domains and impairs the daily life of both patients and caregivers. **Objectives:** To assess the impact of hemophilia A without inhibitors on humanistic outcomes in adult and young patients and their caregivers in a real-life setting. **Methods:** This was a 12-month multicenter prospective observational study conducted in 18 Spanish hospitals. Patients who were diagnosed with hemophilia A (PWHs), without inhibitors, and who were 12 years of age or older, and their caregivers were included in the study. **Results:** A total of 85 PWHs (mean age: 33 years) and 12 caregivers participated in the study; 51 PWHs completed it, representing a 40% lost-to-follow-up rate. Twenty-five percent of PWHs showed maladjustment at study completion, with ‘leisure time’ and ‘work/studies’ being the most affected domains. Quality of life was particularly impaired in the sport, physical health and future areas. ‘Lying/sitting/kneeling/standing’ and ‘leisure activities and sports’ were the most impaired functions. Productivity was mainly affected by presenteeism in adult PWHs; 40 painful joint bleeding episodes were reported. Active strategies were mostly used for coping with chronic pain, and anxiety and/or depression were present in more than 10% of PWHs. Anxiety and depression were more frequently reported by caregivers. **Conclusions:** The study results suggest sustained impairments in adaptation to their disease, quality of life, and functionality in PWHs without inhibitors, especially related to leisure and sports activities, and a nontrivial proportion of them presented clinical levels of depression and anxiety. Overall impairment was more marked in adults than in children. In addition, due to the limited number of caregivers, their results must be considered exploratory.

## 1. Introduction

Hemophilia A (HA) and B (HB) are rare X-linked bleeding disorders due to coagulation factor VIII (FVIII) (in HA) or Factor IX (in HB) gene mutations, with HA being more common (1 in 5000 males) than HB (1 in 30,000 males). Severity is associated with factor levels: severe (<1%), moderate (1–5%), and mild (>5–<40%). Severe forms (53%) cause spontaneous bleeding into joints or muscles, in the absence of identifiable trauma; in moderate forms (19%) there is occasional spontaneous bleeding, while prolonged bleeding occurs primarily after minor trauma or surgery; and, finally, mild forms (28%) are clinically characterized by severe bleeding with major trauma or surgery, although spontaneous bleedings are very rare [1,2]. Recurrent joint bleeding (hemarthrosis), the hallmark symptom of severe hemophilia, has been shown over time to lead to the development of chronic inflammatory, degenerative, and irreversible joint damage known as hemophilic arthropathy, without appropriate prophylactic treatment [3,4], and even sometimes despite administering the right prophylactic therapy [4,5], within a treatment landscape that has evolved significantly in recent years. Replacement therapy with the traditional standard-half-life VIII has been improved with the recently developed extended-half-life FVIII. In addition, the emergence of novel therapies, such as bispecific monoclonal antibodies that mimic FVIII (hemicizumab), which bypass the need for FVIII itself, and rebalancing agents (like Fitusiran) that reduce antithrombin levels through RNA interference, has contributed to decreasing the burden of treatment by lowering bleeding rates and avoiding the need for frequent intravenous infusions, as well as significantly improving patient quality of life [6,7]. Importantly, this complication results in chronic, incapacitating joint pain, compromised health-related quality of life (HRQoL), functional impairment, interference with daily activities, psychosocial disability, and increased onset of anxiety and depression in patients with hemophilia (PWHs) [8,9,10,11,12,13,14,15,16,17,18]. Furthermore, the disease has a negative impact on work life and employment in adult PWHs [12,18,19]. Likewise, hemophilia imposes a significant psychosocial and economic burden on caregivers of PWHs [11,15,16,17,19].

A critical aspect that has been less studied regarding PWHs is how they cope with and adapt to various long-lasting hemophilia-related physical, psychological, and social problems [20,21,22].

The HEMOLIFE study was a prospective study that evaluated the impact of hemophilia A without inhibitors on humanistic outcomes in adult and young patients and their caregivers in a real-life setting in Spain. Among the study outcomes, the following were included: psychological adjustment, behavior, HRQoL, functionality, productivity, strategies for coping with pain, and anxiety and depression. A cross-sectional analysis of the baseline data in this study has been published elsewhere [23].

## 2. Methods

### 2.1. Design

This multicenter, prospective, observational study was conducted in 18 hospitals in Spain by hematologists. The study was approved by the ethics committee of each investigational site, and written informed consent was obtained from each participant.

### 2.2. Study Population

To be included in the study, patients had to be diagnosed with moderate to severe hemophilia A without inhibitors, be 12 years of age or older (comprising children, 12–17 years, and adults, ≥18 years), be able to comply with the study activities, and have signed an informed consent form. Subjects with other coagulopathies or hemostatic disorders were excluded from the study.

Caregivers, who are defined as people who share the disease burden with the patient, were included in the study if they were 18 years of age or older; cared for patients included in the study, who were at least 12 years old with moderate to severe hemophilia A without inhibitors; provided written informed consent; and were able to comply with the study activities.

### 2.3. Study Assessments

The primary objective of this study was to describe the social and occupational impact of hemophilia A in patients without inhibitors at baseline and at 12 months. The secondary study objectives included sociodemographic and clinical descriptions of PWHs, HRQoL assessment, evaluation of loss of work or school days, work productivity, functional assessment, evaluation of pain and strategies to cope with pain, and evaluation of anxiety and depression status.

In addition to the medical records used in our study, we developed a patient-centered healthcare mobile application (app) that allows patients to collect all the necessary study variables throughout the entire duration of the study and enables patients to become the primary data source.

The following information was recorded at baseline via an electronic case report form from medical records: sociodemographic data (including age, sex, and occupational status), medical history, comorbidities, concomitant medications, and clinical variables related to hemophilia.

The humanistic outcomes were assessed during the entire study period using the following validated questionnaires and scales: the Maladjustment Scale [24]; the Hemophilia-Specific Quality of Life Questionnaire for Adults (HaemoQol) [25]; the HaemoQol Short Form for children, with the age upper limit of 16 years [26]; the EQ-5D-5L [27] for HRQoL assessment; the hemophilia-specific version of the Work Productivity and Impairment Questionnaire (WPAI) plus the Classroom Impairment Questionnaire (WPAI+CIQ:HS) [28]; the Hemophilia Activity List (HAL) [29]; the Pediatric Haemophilia Activities List (pedHAL) [30]; the Visual Analog Scale (VAS) for evaluating pain; the Coping Pain Questionnaire-Reduced (CAD-R) [31]; and the Hospital Anxiety and Depression Scale (HADS) [32,33]. The Maladjustment Scale and CAD-R questionnaire were developed and validated in Spain and are described in Table S1. All the collected data were recorded using the app.

The Maladjustment Scale was selected because it assesses the level of negative interference with the main life areas caused by the disease. Although it has not been validated in the hemophilia population, this scale was chosen for our study because it had shown a high internal consistency and good discriminant and concurrent validity for the Spanish general population, as well as sensitivity to change [24]. The Maladjustment Scale consists of a brief 6-item scale that evaluates the degree of maladjustment in five areas of daily life (work, social life, free time, couple relationship, family life), by means of a 6-point Likert scale (from 0 = none to 5 = very much). Higher scores indicate a higher degree of maladjustment in those daily life areas. Total score ranges from 0 to 30. The cutoff points proposed by the authors for maladjustment are a total score ≥ 12 and individual item scores ≥ 2 for each of the six items. A detailed explanation of the CAD-R questionnaire is provided in the Supplementary Information (Table S1). All other questionnaires and scales used in the present study evaluating HRQoL, functional impairment, and pain perception (such as the HaemoQol, HaemoQol Short Form, EQ-5D-5L, WPAI+CIQ: HS, HAL, and pedHAL, and VAS for pain) are validated and widely known assessment tools, most of which are widely used in hemophilia studies [20,32,34,35,36].

### 2.4. Statistical Analysis

Due to the lack of previous information on this scale in PWHs, the study sample size was calculated based on the results obtained from the Maladjustment Scale from a study that evaluated its psychometric properties in patients with psychological disorders and the general population [24]. According to these authors, 100 patients with hemophilia A would allow us to estimate a mean (±standard deviation) value of 3.56 ±1.08 with a 95% confidence interval and a margin of error of 0.21. The study participants were recruited through convenience sampling.

The statistical analyses were mainly descriptive. Quantitative variables are presented as the means and standard deviations or, if applicable, as medians and interquartile ranges. Categorical variables are described using absolute and relative frequencies.

Baseline and 12-month scores (individual items and total scores) from the Maladjustment Scale were compared with Wilcoxon signed-rank test for paired data. The significance level was set at 0.05. In addition, the Cronbach’s alpha for the Maladjustment Scale was calculated to test its reliability since the scale has not been previously validated in the PWH population.

All the analyses were performed with IBM SPSS Statistics V26.

## 3. Results

### 3.1. Study Participants

From June 2020 to April 2022, 85 PWHs from 18 participating sites were enrolled in this study, with the study population being below the estimated sample size of 100 PWHs. The study subjects had a mean (SD) age of 33 (14) years, with 80% of them being at least 18 years of age. Thirty-two (38%) PWHs were married or living with a partner, and 37 (44%) were employed. A high percentage of the study patients (83%) were diagnosed with severe hemophilia A, with a mean (SD) time since diagnosis of 342 (168) months, and 41 (48%) presented with comorbidities, although only 14 (16.5%) presented with hemophilic arthropathy. Seventy-seven PWHs (90%) received prophylactic treatment for hemophilia prior to their inclusion in the study. All the sociodemographic and clinical characteristics are displayed in Table 1. At the end of the study period (12 months), 51 PWHs continued in the study, which represents a 40% loss-to-follow-up. A post hoc analysis has shown that there were baseline statistical differences between completers (*n* = 51) and non-completers (*n* = 34), respectively, in two variables: age, years (mean ± standard deviation) of 35.7 (13.3) versus 29.1 (13.8), *p* < 0.05; and presence of comorbidities (*n*, %) of 31 (60.8) versus 10 (29.4), *p* < 0.001. A detailed comparison of all the baseline characteristics in completers and non-completers is shown in the Supplementary Table S2.

Additionally, 6 (7%) PWHs received social assistance, and 12 (14%) received support from a caregiver. Among the 12 caregivers, 10 (80%) were female, and six (50%) were either mothers or fathers. Eight (67%) were employed (including three caregivers who were self-employed).

### 3.2. Maladjustment Evaluation

**Figure 1:**
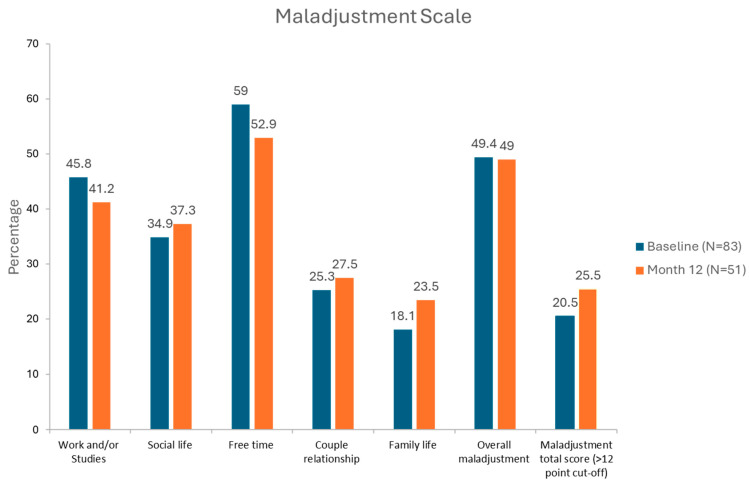
Maladjustment Scale.

The mean (SD) total score values of the Maladjustment Scale were 7.5 (7.0) and 7.7 (8.0) at baseline and at 12 months, respectively (*p* = 0.232). Among the 83 evaluated subjects at baseline, 17 (20.5%) exhibited maladjustment, whereas at study completion, 13 of the 51 subjects (25.5%) showed maladjustment (Figure 1). However, these differences were not statistically significant (*p* = 0.72). The individual areas of life most affected by maladjustment, either at baseline or at 12 months, were ‘leisure time’ (59% and 53%, respectively) and ‘work/studies’ (46% and 41%, respectively). In contrast, the least affected area was ‘family life,’ with 18% and 23.5% at baseline and at the 12-month evaluation, respectively. Almost 50% of PWHs reported the interference of hemophilia with their daily lives (Figure 1). There were no significant differences between baseline and month 12 in the proportion of maladjustment in any of the dimensions of the Maladjustment Scale (*p* values not shown).

The cutoff points for maladjustment are a total score ≥ 12 and individual item scores ≥ 2 for each of the six items.

The Cronbach’s alpha for the Maladjustment Scale in the present study was 0.938, confirming the high reliability of the tool.

### 3.3. Quality-of-Life Assessment

The HaemoQoL questionnaire in adult PWHs yielded the greatest impairment in the ‘sport’, ‘physical health’ and ‘future’ domains, with baseline mean (SD) transformed scores of 49.6 (28.2), 39.8 (25.8), and 37.3 (28.8), respectively, and mean (SD) transformed scores at study completion of 58.4 (26.9), 42.8 (28.4), and 40.5 (26.2), respectively (Table 2).

In the pediatric population, as assessed with the Haemo-QoL-SF for children and adolescents, the most affected domains in the youngest group (12 years old) at baseline were a perception of annoyance of the injections, with a mean (SD) transformed score of 50 (7.1), and friends and social support, with the same mean (SD) value of 34.4 (4.4). No results were available in this group at the 12-month evaluation (Table 2). For the adolescent group (13 to 16 years old), the greatest impairment was reported in the dimensions of health, social support and the future, with baseline mean (SD) normalized scores of 31.8 (28.6), 31.8 (28.6), and 28.1 (15.4), respectively, and 12-month mean (SD) scores of 33.0 (29.5), 33.0 (29.5), and 37.5 (33.8), respectively (Table 2).

### 3.4. Assessment of Functional Ability

Among the adult PWHs, the most impaired functions at baseline and at study completion were ‘function of legs,’ ‘lying/sitting/kneeling/standing,’ and ‘leisure activities and sports,’ with baseline mean (SD) normalized scores of 63.8 (26.9), 64.9 (25.4), and 71.0 (25.9), respectively, and 12-month mean (SD) values of 61.7 (27.2), 59.64 (26.5), and 67.4 (24.8), respectively. The least affected function in adults was self-care, with mean (SD) scores of 87.8 (19.7) at baseline and 81.9 (26.3) at 12 months (Table 3). In the pediatric population, all dimensions were comparable in their degree of impairment, with mean normalized scores ranging from 82.7 (21.7) (‘Leisure activities and sports) to 92.7 (21.2) (‘Self-care’) at baseline and from 90.2 (22.0) for ‘Leisure activities and sports’ to 100 (0.0) for Household tasks after twelve months of follow-up (Table 3).

### 3.5. Productivity Evaluation

At baseline, 58 PWHs were assessed for productivity: 32 (39.5%), with a median age (Q1, Q3) of 35.5 years (31.5, 46.0), were employed (as per the WPAI+CIQ:HS definition of working for pay), and 26 (32%), with a median age (Q1, Q3) of 17.5 years (15.0, 27.0), were students, although nine PWHs completed both questionnaires, the work-related and the classroom/academic one. At study completion, 20 employed PWHs and 13 students were evaluated for productivity. At the beginning of the study, the average work productivity of the 29 evaluable subjects, which is defined as the mean percentage of total hours worked in the previous week divided by the total number of hours worked, was 94.4. The mean percentage was reduced to 85.4 at the end of the study.

PWHs who were employed reported a greater proportion of presenteeism (i.e., impaired work productivity due to a health problem) than absenteeism (i.e., missed work time due to a health problem), both at baseline (20.9% and 5.6%, respectively) and at study completion (21.6% and 14.8%, respectively) (Table 4). Among the students, the mean percentage of class time missed due to hemophilia was 4.9%, and the mean percentage of impairment in the classroom was 10.8% at baseline; the corresponding percentages at month 12 were 10.3% and 8.5%, respectively (Table 4). In the overall evaluable population (*n* = 80), the mean percentage of activity impairment was 24.1% at baseline and 29.6% at the end of the study period (Table 4).

### 3.6. Pain Perception and Evaluations of Coping Strategies for Chronic Pain

In this study, painful bleeding episodes in joints were reported by 40 PWHs, with a mean (SD) VAS score of 40.3 (26.4) at the end of the study.

Regarding chronic pain coping strategies, as assessed with the CAD-R scale, PWHs reported higher mean score values for active face-off strategies (i.e., distraction, mental self-control, self-affirmation, and the search for information) than for passive strategies (i.e., religion and catharsis). Overall, the mean CAD-R scores were similar at baseline and at the end of the study (Table 5).

In addition, 41 PWHs (48% of the entire study population) were taking medications for pain control.

### 3.7. Anxiety and Depression Assessment

At baseline, 9 (11.1%) of the 81 evaluable patients experienced clinically significant anxiety, and 9 (11.1%) experienced clinically significant depression. Furthermore, at the end of the study, there was an increase in the percentage of PWHs with clinically significant anxiety (13 of 51 PWHs [25.5%]), whereas the proportion of PWHs with clinically significant depression (5 of 51 PWHs [9.8%]) was similar to that at baseline.

### 3.8. Caregivers’ Evaluation of Quality of Life, Work Productivity, and Anxiety/Depression

Results of caregivers’ assessment of HRQoL (using the EQ-5D-5L), work productivity (evaluated with the WPAI-GH), and anxiety/depression (according to the HADS) are shown in Supplementary Table S3.

## 4. Discussion

In this prospective longitudinal study, we observed that one-fourth of PWHs showed maladjustment behavior, and this proportion seemed to be maintained after 12 months of follow-up. The most impaired areas of daily life are leisure time, sports, and work/studies, which is compatible with the fact that most hemophilia-related hemorrhages occur in the musculoskeletal system [37]. To the best of our knowledge, this study is the first to assess the degree of maladjustment in PWH using this scale. We found a higher mean degree of maladjustment compared with a normative population (mean score of 7.70 in the PHW population vs. 2.22 in the normative population) [24]) and a lower degree of maladjustment than did a population of people affected by different mental disorders (mean score of 18.0) [24].

We observed an age-related pattern of impaired quality of life. While the adult subpopulation regarded physical health and sport activities as the most negatively impacted dimensions, younger PWHs (13–16 years of age), in turn, considered friendship and social interaction behaviors the most affected areas, observations that could be explained by the critical sense of belonging to a peer group among young patients with hemophilia (10 to 24 years old) [38]. The most negatively impacted QoL domains in the adult segment of our study population, such as ‘physical health’ and ‘sports,’ are consistent with the findings reported in the Maladjustment Scale. Additionally, the overall quality-of-life results in our study are consistent with those reported in other published studies of PWHs, in spite of some methodological differences, such as study designs, populations and study outcomes. Children with hemophilia have been shown to have greater impairment in the ‘perceived support’ and ‘friends’ domains [34,39]. Likewise, studies evaluating HRQoL results using the Haemo-QoL questionnaire among adult PWHs have revealed that ‘physical health,’ ‘sports,’ and ‘perception of the future’ were the most affected areas [39,40], supporting our findings. Other studies have yielded similar QoL results using different HRQoL evaluation tools [41,42]. Interestingly, an Italian research group has demonstrated that an increased knowledge of hemophilia among sports physicians, along with the right prophylaxis treatment for PWHs, would allow these patients to be included in sports groups and physical activity programs, thus contributing to improving their HRQoL [43,44].

Consistent with what we have described for maladjustment and quality of life, the most impaired domains in the evaluation of functioning with the HAL in the adult subpopulation were ‘lying/sitting/kneeling/standing,’ ‘function of legs,’ and ‘leisure activities and sports.’ With respect to the child subpopulation (12 to 17 years of age), the lowest scores were also observed in some domains, such as ‘lying/sitting/kneeling/standing’ and ‘leisure activities and sports,’ albeit with values greater than 80. We observed a worse functional status in the adult PWH group than in the child subpopulation, which is also consistent with the quality-of-life findings from the Haemo-QoL. Our results are in line with other published observations in adult PWHs, where the most affected HAL domains were also ‘lying/sitting/kneeling/standing’ and ‘function of legs’ [41,45]. Greater functional impairment among adult PWHs might be due to the progressive deterioration of the disease over time [8,46,47], sometimes despite correct prophylaxis treatment [5]. In addition, it is important to highlight that the least affected domain observed in our study was self-care, which is in accordance with a greater adaptation of the PWHs to the family setting rather than to the social and work environments, as reported in the literature [45,48].

Similarly, we observed a greater negative effect of the disease on working PWH productivity than on student PWH productivity. The results from the WPAI+CIQ:HS questionnaire revealed that, at baseline, productivity in the working patients (median age of 35.5 years) was more strongly impaired by presenteeism than by absenteeism. Likewise, student productivity, assessed in a younger population of PWHs, with a median age of 17.5 years, seems to be more affected by presenteeism than by absenteeism, although to a lesser extent than that of the working PWHs. In our view, parenteral overprotection may partly explain the increased impairment in the productivity of young students with hemophilia. The results from the few studies that have investigated work productivity in adult PWHs using the WPAI questionnaire support our findings [10,32,49].

Although the frequency of hemophilic arthropathy in our study sample was low (16.5%), painful bleeding episodes into the joints were reported by almost half of the study population throughout the follow-up period. In the present study, we observed that PWHs use the most commonly active strategies for coping with chronic pain, namely, distraction, searching for information, and self-control. This is an important finding because active cognitive and behavioral strategies, such as problem solving, social support, and information seeking, have been associated with the most effective and ready disposition to self-manage pain and with quality of life improvement, whereas passive pain coping strategies and negative thoughts may impair mental quality of life [20].

The proportion of patients with either depression or anxiety at the beginning of our study was relevant (over 10%) but lower than the prevalence reported in other studies on hemophilia, ranging from 28% [17] to 41% [41]. To some extent, this finding is consistent with the good general health status of the study population and the low percentage of patients with hemophilic arthropathy.

This study has several limitations that must be acknowledged. Our primary outcome was evaluated using an evaluation tool that has not been validated in PWHs because we are not aware of any validated tool for evaluating this dimension in PWHs. However, through a post hoc evaluation, the Maladjustment Scale has shown good internal consistency in our sample of PWHs, as evaluated with Cronbach’s alpha, even though it does not constitute formal validation. Furthermore, cut-off points derived from populations cannot be directly extrapolated to the hemophilia clinical context. Another limitation was that the sample size calculation was based on a population that was not clinically comparable to PWHs, and, moreover, the planned sample size was not reached, which could have led to imprecise results. The main reason for not reaching the planned sample size was the COVID-19 pandemic, along with the consequent reduction in in-person outpatient care services. The study was conducted in a single country, which limits its generalizability. Additionally, our study was primarily descriptive and did not incorporate any predefined longitudinal analytical strategy that might lead to inferential conclusions. Moreover, relationships among the study variables could not be drawn since these analyses were not established in the study protocol and were not performed. Finally, we must assume that a relevant proportion of patients were lost in follow-up (≥40%), mostly due to the COVID-19 pandemic, with the consequent high risk of attrition bias and further limiting generalizability. Nonetheless, there were only significant differences in two baseline characteristics (age and comorbidities) between completers and non-completers at study completion. PWHs who completed follow-up were older and exhibited more comorbidities than those who did not complete follow-up. This may have affected the outcomes. Although it is difficult to establish the direction of the effect, it is likely that PWHs with more comorbidities show a higher burden and worse psychosocial outcomes.

## 5. Conclusions

Our results suggest that patients with hemophilia A without inhibitors present substantial and sustained impairments in adjusting to their disease, quality of life, and functionality, especially related to leisure and sports activities, and a relevant proportion of these patients exhibit clinical levels of depression and anxiety. Impairment is more marked in adults than in children. Furthermore, adult PWHs seem to be more aware of their disease, whereas younger patients appear to carry a rather normal life, with less disease impact on their daily activities, most likely due to new improvements in medical treatment [50]. These observations, recorded at the end of the longitudinal study follow-up, suggest the maintenance of the published baseline results [23]. In addition, our results also suggest a relevant impairment in the psychological well-being of caregivers, although these findings are exploratory in nature and should be interpreted with caution, due to their small sample size. Our study results are in accordance with the need to improve hemophilia management in terms of both disease detection and prophylactic treatment, and support the necessity of a holistic approach to managing PWHs, thus placing the disease directly within the framework of the biopsychosocial model, which integrates the interconnected biological, psychological, and social dimensions of health and disease [51,52]. Moreover, research evaluating interventions for improving HRQoL, psychological well-being, and coping with the disease is needed because it seems to be a neglected area of research [52].

## Figures and Tables

**Table 1:** Demographic and clinical characteristics.

Characteristic	N	
**Age (years)**	85	
Mean ± SD		33.1 (13.8)
12 to <18 years, *n* (%)		17 (20.0)
18 to <35 years, *n* (%)		31 (36.5)
≥35 years, *n* (%)		37 (43.5)
**Race, *n* (%)**	85	
Caucasian		75 (88.2)
Other		10 (11.8)
**Marital status, *n* (%)**	84	
Married/with a partner		32 (38.1)
Single		48 (57.1)
Divorced		4 (4.8)
**Employment status, *n* (%)**	84	
Employed (or self-employed)		37 (44.0)
Not employed		47 (56.0)
**Education level (college or above ^a^** **), *n* (%)**	84	29 (34.5)
**Body mass index (kg/m^2^), mean (SD)**	80	25.6 (6.3)
**Hemophilia severity, *n* (%)**	85	
Moderate		14 (16.5)
Severe		71 (83.5)
**Time since diagnosis of hemophilia (months), mean (SD)**	53	342.7 (168.6)
**Age at diagnosis of hemophilia (years),** **mean (SD)**	53	3.5 (8.5)
**Treatment (prophylaxis) for hemophilia, *n* (%)**	85	77 (90.6)
**Comorbidities ^b^** **, *n* (%)**	85	
Any		41 (48.2)
Hemophilic arthropathy		14 (16.5)
Hepatitis C		10 (11.8)
HIV		8 (9.4)
Hypertension		8 (9.4)
Obesity		4 (4.7)
Hypercholesterolemia		4 (4.7)
Diabetes		4 (4.7)
**Social assistance for hemophilia *n* (%)**	84	6 (7.1)
**Caregiver ^c^** **(yes), *n* (%)**	85	12 (14.1)

^a^ Including a university degree, master’s degree or doctorate; ^b^ most frequent (≥4.5%) comorbidities; ^c^ three caregivers were reported by patients, but they did not sign the informed consent form; hence, they did not participate in the study.

**Table 2:** Quality of life in persons with hemophilia A according to the HaemoQoL results.

	Baseline			Month 12	
Dimension Score (Transformed ^b^), Mean (SD) ^c^	Adults N = 66	Children 12 y N = 2	Children 13–16 y ^a^ N = 12	Adults N = 44	Children 13–16 y N = 7
Physical health (injections ^d^)	-	50.0 (7.1)	22.5 (23.8)	-	14.3 (19.7)
Physical health	39.8 (25.8)	30.4 (7.6)	19.6 (18.5)	42.8 (28.4)	10.7 (13.0)
Feeling	20.3 (24.8)	21.4 (5.1)	20.1 (24.1)	25.0 (26.2)	6.7 (14.0)
View	29.9 (22.8)	31.9 (21.6)	24.4 (19.1)	33.8 (25.4)	12.1 (10.7)
Family	-	10.0 (14.1)	25.5 (18.1)	-	12.5 (13.4)
Friends	-	34.4 (4.4)	31.8 (28.6)	-	33.0 (29.5)
Social support	-	34.4 (4.4)	31.8 (28.6)	-	33.0 (29.5)
Social compare	-	14.6 (2.9)	18.4 (24.3)	-	7.6 (10.7)
Sports	49.6 (28.2)	12.5 (13.3)	22.0 (18.0)	58.4 (26.9)	15.3 (16.8)
Work	20.1 (26.2)	-	-	25.0 (27.2)	-
Hemophilia management	24.5 (25.5)	35.7 (5.1)	26.8 (20.2)	25.2 (26.1)	29.2 (36.7)
Treatment	33.3 (21.5)	16.1 (12.6)	19.8 (15.8)	34.7 (24.3)	20.8 (16.3)
Future	37.3 (28.8)	-	28.1 (15.4)	40.5 (26.2)	37.5 (33.8)
Family planning	12.1 (15.7)	-	-	14.2 (20.7)	-
Partnership	11.6 (20.1)	-	8.3 (15.4)	12.3 (24.1)	2.1 (5.1)
Total score	29.9 (17.0)	28.6 (1.5)	22.3 (12.7)	33.3 (17.6)	14.2 (12.4)

^a^ 16 years old is the highest age category for children/adolescents. ^b^ Transformed scores range from 0 to 100, with high scores (near 100) indicating low quality of life. ^c^ Reported as the proportion of patients who answered excellent/very good/good. ^d^ Perception of annoyance of the injections. “-”, Not available. SD, standard deviation; y, years.

**Table 3:** Functional assessment of PWHs.

	Baseline				Month 12			
Hemophilia Activities List (HAL) Domain, Normalized Scores ^a^	Adults (≥18 years)		Children (≥12 to <18 years)		Adults (≥18 years)		Children (≥12 to <18 years)	
	N	Mean (SD)	N	Mean (SD)	N	Mean (SD)	N	Mean (SD)
Lying/sitting/kneeling/standing	66	64.9 (25.4)	14	84.5 (22.1)	44	59.64 (26.5)	5	93.9 (12.3)
Function of the legs	66	63.8 (26.9)	14	85.8 (23.1)	44	61.7 (27.2)	5	91.3 (18.5)
Function of the arms	66	78.0 (24.0)	14	88.6 (20.0)	44	74.1 (25.3)	5	98.7 (3.0)
Use of transportation	64	76.1 (27.1)	14	89.0 (23.3)	39	71.4 (30.5)	5	98.7 (3.0)
Self-care	66	87.8 (19.7)	14	92.7 (21.2)	44	81.9 (26.3)	5	99.6 (1.0)
Household tasks	66	80.5 (25.7)	14	87.6 (24.2)	44	76.5 (26.8)	5	100 (0.0)
Leisure activities and sports	60	71.0 (25.9)	14	82.7 (21.7)	44	67.4 (24.8)	5	90.2 (22.0)
Total score	66	72.4 (21.8)	14	86.7 (20.5)	44	68.4 (23.3)	5	94.7 (11.4)

^a^ Normalized scores range from 0 to 100, where higher scores represent better functional status. PWHs, persons with hemophilia; SD, standard deviation.

**Table 4:** Work productivity according to the WPAI-CIQ:HS.

	Baseline		Month 12	
WPAI-CIQ Variable	N *	Mean (SD)	N	Mean (SD)
Percent work time missed	29	5.6 (19.0)	19	14.8 (29.3)
Percent impairment while working	32	20.9 (29.8)	19	21.6 (27.3)
Percent overall work impairment	29	20.8 (28.0)	19	26.5 (34.9)
Percent class time missed	26	4.9 (11.6)	12	10.3 (22.8)
Percent impairment in the classroom	26	10.8 (17.9)	13	8.5 (20.3)
Percent overall classroom impairment	26	14.1 (21.3)	12	14.3 (30.2)
Percent regular activity impairment	80	24.1 (27.9)	49	29.6 (29.0)

* Nine patients completed both work-related and classroom/academic questions from the WPAI-CIQ:HS. SD, standard deviation; WPAI-CIQ: HS, Work Productivity and Activity Impairment Questionnaire plus Classroom Impairment Questionnaire: Hemophilia Specific.

**Table 5:** Coping strategies for chronic pain.

	Baseline		Month 12	
Factors of the CAD-R	N	Mean (SD)	N	Mean (SD)
Distraction	83	9.4 (3.3)	51	9.8 (3.4)
Search for information	83	9.5 (4.1)	51	9.0 (3.8)
Religion	83	6.1 (4.0)	51	6.0 (3.8)
Catharsis	83	8.3 (3.6)	51	7.5 (2.9)
Mental self-control	83	9.3 (4.2)	51	9.1 (4.1)
Self-affirmation	83	13.9 (4.2)	51	13.7 (4.4)
Second order factors—Active pain face-off	83	42.1 (11.2)	51	41.6 (10.3)
Second order factors—Passive pain face-off	83	14.4 (6.6)	51	13.4 (5.6)

CAD-R, Coping Pain Questionnaire-Reduced; SD, standard deviation.

## Data Availability

The data that support the findings of this study are available from the corresponding author upon reasonable request.

## Supplementary Materials

The following supporting information can be downloaded at https://www.mdpi.com/article/10.3390/jcm15051790/s1: Table S1. Description of the CAD-R questionnaire. Table S2. Comparison of baseline characteristics between completers and non-completers. Table S3. Caregivers’ Evaluation of Quality of Life, Work Productivity, and Anxiety/Depression.

## References

[1] Castaman G., Matino D. (2019). Hemophilia A and B: Molecular and clinical similarities and differences. Haematologica.

[2] Srivastava A., Santagostino E., Dougall A., Kitchen S., Sutherland M., Pipe S.W., Carcao M., Mahlangu J., Ragni M.V., Windyga J. (2020). WFH Guidelines for the Management of Hemophilia, 3rd edition. Haemophilia.

[3] Knobe K., Berntorp E. (2011). Haemophilia and joint disease: Pathophysiology, evaluation, and management. J. Comorbidity.

[4] Gualtierotti R., Solimeno L.P., Peyvandi F. (2021). Hemophilic arthropathy: Current knowledge and future perspectives. J. Thromb. Haemost..

[5] De la Corte-Rodríguez H., Bystrická L., Ball N., Olsen S., Golden K., Hakimi Z., Kragh N. (2024). Assessment of joint health in patients receiving prophylaxis for haemophilia A: A cross-sectional survey in five European countries. BMJ Open.

[6] Reding M.T. (2025). New therapies in hemophilia: Extend the half-life, mimic, or rebalance?. Hematology Am. Soc. Hematol. Educ. Program..

[7] Gogia P., Tarantino M., Schramm W., Aledort L. (2023). New directions to develop therapies for people with hemophilia. Expert Rev. Hematol..

[8] Blokzijl J., Pisters M.F., Veenhof C., Schutgens R.E.G., Timmer M.A. (2023). Functional decline in persons with haemophilia and factors associated with deterioration. Haemophilia.

[9] Cavazza M., Kodra Y., Armeni P., De Santis M., López-Bastida J., Linertová R., Oliva-Moreno J., Serrano-Aguilar P., Posada-de-la-Paz M., Taruscio D. (2016). Social/economic costs and quality of life in patients with haemophilia in Europe. Eur. J. Health Econ..

[10] Curtis R., Manco-Johnson M., Konkle B.A., Kulkarni R., Wu J., Baker J.R., Ullman M., Tran D.Q., Nichol M.B. (2022). Comorbidities, health-related quality of life, health-care utilization in older persons with hemophilia—Hematology utilization group study part VII (HUGS VII). J. Blood Med..

[11] Fornari A., Antonazzo I.C., Rocino A., Preti D., Fragomeno A., Cucuzza F., Ceresi N., Santoro C., Ferretti A., Facchetti R. (2024). The psychosocial impact of haemophilia from patients’ and caregivers’ point of view: The results of an Italian survey. Haemophilia.

[12] Forsyth A.L., Witkop M., Lambing A., Garrido C., Dunn S., Cooper D.L., Nugent D.J. (2015). Associations of quality of life, pain, and self-reported arthritis with age, employment, bleed rate, and utilization of hemophilia treatment center and health care provider services: Results in adults with hemophilia in the HERO study. Patient Prefer. Adherence.

[13] Kempton C.L., Buckner T.W., Fridman M., Iyer N.N., Cooper D.L. (2018). Factors associated with pain severity, pain interference, and perception of functional abilities independent of joint status in US adults with hemophilia: Multivariable analysis of the pain, functional impairment, and quality of life (P-FiQ) study. Eur. J. Haematol..

[14] Lorenzato C.S., Santos R.B., Fagundes G.Z.Z., Ozelo M.C. (2019). Haemophilia experiences, results and opportunities (HERO study) in Brazil: Assessment of the psychosocial effects of haemophilia in patients and caregivers. Haemophilia.

[15] Ramos-Petersen L., Rodríguez-Sánchez J.A., Cortés-Martín J., Reinoso-Cobo A., Sánchez-García J.C., Rodríguez-Blanque R., Coca J.R. (2023). A qualitative study exploring the experiences and perceptions of patients with hemophilia regarding their health-related well-being, in Salamanca. J. Clin. Med..

[16] Recht M., Neufeld E.J., Sharma V.R., Solem C.T., Pickard A.S., Gut R.Z., Cooper D.L. (2014). Impact of acute bleeding on daily activities of patients with congenital hemophilia with inhibitors and their caregivers and families: Observations from the Dosing observational study in hemophilia (DOSE). Value Health.

[17] Carlsson K.S., Winding B., Astermark J., Baghaei F., Brodin E., Funding E., Holmström M., Österholm K., Bergenstråle S., Andersson E. (2022). Pain, depression and anxiety in people with haemophilia from three Nordic countries: Cross-sectional survey data from the MIND study. Haemophilia.

[18] Witkop M., Guelcher C., Forsyth A., Hawk S., Curtis R., Kelley L., Frick N., Rice M., Rosu G., Cooper D.L. (2015). Treatment outcomes, quality of life, and impact of hemophilia on young adults (aged 18–30 years) with hemophilia. Am. J. Hematol..

[19] Cutter S., Molter D., Dunn S., Hunter S., Peltier S., Haugstad K., Frick N., Holot N., Cooper D.L. (2017). Impact of mild to severe hemophilia on education and work by US men, women, and caregivers of children with hemophilia B: The bridging hemophilia b experiences, results and opportunities into solutions (B-HERO-S) study. Eur. J. Haematol..

[20] Elander J., Robinson G., Mitchell K., Morris J. (2009). An assessment of the relative influence of pain coping, negative thoughts about pain, and pain acceptance on health-related quality of life among people with hemophilia. Pain.

[21] Canclini M., Saviolo-Negrin N., Zanon E., Bertoletti R., Girolami A., Pagnan A. (2003). Psychological aspects and coping in haemophilic patients: A case-control study. Haemophilia.

[22] Cuesta-Barriuso R., Torres-Ortuño A., Nieto-Munuera J., López-Pina J.A. (2021). Quality of life, perception of disease and coping strategies in patients with hemophilia in spain and el salvador: A comparative study. Patient Prefer. Adherence.

[23] Álvarez-Román M.T., Nuñez Vazquez R.J., Benitez Hidalgo O., Quintana Paris L., Entrena Ureña L., Lopez Jaime F.J., la De Corte-Rodríguez H., García Dasí M., Bosch P., Mingot Castellano M.E. (2024). Humanistic burden of haemophilia A without inhibitors: A cross-sectional analysis of the HemoLIFE study. Haemophilia.

[24] Echeburúa E., Gargallo P., Fernández-Montalvo J. (2000). Escala de inadaptación (EI): Propiedades psicométricas en contextos clínicos. Anál. Modif. Conducta.

[25] Rentz A., Flood E., Altisent C., Bullinger M., Klamroth R., Garrido R.P., Scharrer I., Schramm W., Gorina E. (2008). Cross-cultural development and psychometric evaluation of a patient-reported health-related quality of life questionnaire for adults with haemophilia. Haemophilia.

[26] Pollak E., Mühlan H., Von Mackensen S., Bullinger M. (2006). The Haemo-QoL index: Developing a short measure for health-related quality of life assessment in children and adolescents with haemophilia. Haemophilia.

[27] Herdman M., Gudex C., Lloyd A., Janssen M., Kind P., Parkin D., Bonsel G., Badia X. (2011). Development and preliminary testing of the new five-level version of EQ-5D (EQ-5D-5L). Qual. Life Res..

[28] Minno G., Santagostino E., Morfini M., Ettorre C., Cultrera D., Baldacci E., Russo E., Gallucci C. (2019). Work Productivity and Activity Impairment Questionnaire (WPAI) + Classroom Impairment Questions (CIQ): Hemophilia Specific (HS). https://www.semanticscholar.org/paper/Work-Productivity-and-Activity-Impairment-(-WPAI-)-Minno-Santagostino/636fe76b7515c290f2c50e46b2e515701dfde726.

[29] van Genderen F.R., van Meeteren N.L., van der Bom J.G., Heijnen L., de Kleijn P., van den Berg H.M., Helders P.J. (2004). Functional consequences of haemophilia in adults: The development of the haemophilia activities list. Haemophilia.

[30] Kuijlaars I.A.R., van der Net J., Schutgens R.E.G., Fischer K. (2019). The paediatric haemophilia activities list (pedHAL) in routine assessment: Changes over time, child-parent agreement and informative domains. Haemophilia.

[31] Soriano J., Monsalve V. (2017). CAD-R. Cuestionario de afrontamiento al dolor crónico: Análisis factorial confirmatorio.¿ Hay diferencias individuales en sexo, edad y tipo de dolor?. Rev. Soc. Esp. Dolor.

[32] Di Minno G., Santagostino E., Morfini M., Ettorre C., Cultrera D., Baldacci E., Russo E., Gallucci C. (2019). Patient satisfaction and acceptability of an on-demand and on-prophylaxis device for factor VIII delivery in patients with hemophilia A. Patient Prefer. Adherence.

[33] Zigmond A.S., Snaith R.P. (1983). The hospital anxiety and depression scale. Acta Psychiatr. Scand..

[34] Dekoven M., Wisniewski T., Petrilla A., Holot N., Lee W.C., Cooper D.L., von Mackensen S. (2013). Health-related quality of life in haemophilia patients with inhibitors and their caregivers. Haemophilia.

[35] Khair K., Holland M., Bladen M., Griffioen A., McLaughlin P., von Mackensen S. (2017). Study of physical function in adolescents with haemophilia: The SO-FIT study. Haemophilia.

[36] O’Hara J., Walsh S., Camp C., Mazza G., Carroll L., Hoxer C., Wilkinson L. (2018). The impact of severe haemophilia and the presence of target joints on health-related quality-of-life. Health Qual. Life Outcomes.

[37] Rodriguez-Merchan E.C. (2010). Musculoskeletal complications of hemophilia. HSS J..

[38] Königs C., Motwani J., Jiménez-Yuste V., Blatný J. (2024). Teenagers and adolescents with hemophilia-need for a specific approach. J. Clin. Med..

[39] Das S., Roy R.N., Das D.K., Chakraborty A., Mondal R. (2019). Health-related quality of life of hemophilics and its possible correlates: A perspective in health promotion and disability prevention. J. Educ. Health Promot..

[40] Cheung Y.T., Lam P.H., Lam H.H.W., Ma C.T., Leung A.W.K., Wong R.S.M., Li C.K. (2022). Treatment adherence and health-related quality of life in patients with hemophilia in Hong Kong. Int. J. Environ. Res. Public Health.

[41] Buckner T.W., Batt K., Quon D., Witkop M., Recht M., Kessler C., Baumann K., Hernandez G., Wang M., Cooper D.L. (2018). Assessments of pain, functional impairment, anxiety, and depression in US adults with hemophilia across patient-reported outcome instruments in the pain, functional impairment, and quality of life (P-FiQ) study. Eur. J. Haematol..

[42] Carroll L., Benson G., Lambert J., Benmedjahed K., Zak M., Lee X.Y. (2019). Real-world utilities and health-related quality-of-life data in hemophilia patients in France and the United Kingdom. Patient Prefer. Adherence.

[43] Lassandro G., Accettura D., Giordano P. (2021). Promoting Sports Practice in Persons with Hemophilia: A Survey of Clinicians’ Perspective. Int. J. Environ. Res. Public Health.

[44] Lassandro G., Pastore C., Amoruso A., Palladino V., Accettura D., Buzzi A., Tafuri S., Gallone M.F., Valente R., Trisciuzzi R. (2022). Eligibility for competitive sport medical certification of children with severe hemophilia: Italian observational study. Acta Biomed..

[45] van Balen E.C., Haverman L., Hassan S., Taal E.M., Smit C., Driessens M.H., Beckers E.A.M., Coppens M., Eikenboom J., Hooimeijer H.L. (2021). Validation of PROMIS Profile-29 in adults with hemophilia in the Netherlands. J. Thromb. Haemost..

[46] Hodroj M.H., El Hasbani G., Al-Shamsi H.O., Samaha H., Musallam K.M., Taher A.T. (2022). Clinical burden of hemophilia in older adults: Beyond bleeding risk. Blood Rev..

[47] Kuijlaars I.A.R., Timmer M.A., de Kleijn P., Pisters M.F., Fischer K. (2017). Monitoring joint health in haemophilia: Factors associated with deterioration. Haemophilia.

[48] Kuijlaars I.A.R., van der Net J., Buckner T.W., Kempton C.L., Schutgens R.E.G., Fischer K. (2021). Shortening the haemophilia activities list (HAL) from 42 items to 18 items. Haemophilia.

[49] O’Hara J., Noone D., Jain M., Pedra G., Landis S., Hawes C., Burke T., Camp C. (2021). Clinical attributes and treatment characteristics are associated with work productivity and activity impairment in people with severe haemophilia A. Haemophilia.

[50] López-Jaime F.J., Benítez O., Díaz Jordán B.L., Montaño A., Coll J., Quintana París L., Gómez-Del Castillo Solano M.D.C. (2023). Expert opinion paper on the treatment of hemophilia a with emicizumab. Hematology.

[51] Engel G.L. (2012). The need for a new medical model: A challenge for biomedicine. Psychodyn. Psychiatry.

[52] Palareti L., Melotti G., Cassis F., Nevitt S.J., Iorio A. (2020). Psychological interventions for people with hemophilia. Cochrane Database Syst. Rev..

