# Supplementary material for: Psychosocial Outcomes and Quality of Life in Patients with Hemophilia a Without Inhibitors: The HemoLIFE Study
Source: J Clin Med. 2026 Feb 27;15(5):1790. doi: 10.3390/jcm15051790 (PMC12986524; doi:10.3390/jcm15051790)
Supplement: Supplementary file 1 [file jcm-15-01790-s001.zip › jcm-4056958-supplementary.pdf]

Supplementary Materials

Table S1. Description of the CAD-R questionnaire

Coping Pain Questionnaire-Reduced Version (CAD-R)	A questionnaire that evaluates the degree of adjustment to chronic pain. It consists of 24 items grouped under six factors that represent pain coping strategies: self-affirmation, information seeking, religion, distraction, catharsis, and mental self-control; and they are scored with a 5-point Likert scale (from 1=never to 5=always).

Table S2. Comparison of baseline characteristics between completers and non-completers

Characteristic	N		Follow-up completed (12 months)		p-value[a]
			Yes	No	
**Age (years)**	**85**		**51**	**34**	
Mean (SD)		33.1 (13.8)	35.7 (13.3)	29.1 (13.8)	**0.033**
<18 years, n (%)		17 (20.0)	7 (13.7)	10 (29.4)	
18 to <35 years, n (%)		31 (36.5)	18 (35.3)	13 (38.2)	0.123
≥35 years, n (%)		37 (43.5)	26 (51.0)	11 (32.4)	
**Race, n (%)**	**85**		**51**	**34**	
Caucasian		75 (88.2)	44 (86.3)	31 (91.2)	0.733
Other		10 (11.8)	7 (13.7)	3 (8.8)	
**Marital status, n (%)**	**84**		**51**	**33**	
Married/with a partner		32 (38.1)	23 (45.1)	9 (27.3)	
Single		48 (57.1)	3 (5.9)	1 (3.0)	0.173
Divorced		4 (4.8)	25 (49.0)	23 (69.7)	
**Employment status, n (%)**	**84**		**51**	**33**	
Employed (or self-employed)		37 (44.0)	23 (45.1)	14 (42.4)	0.809
Not employed		47 (56.0)	28 (54.9)	19 (57.6)	
**Education level (college or above), n (%)**	**84**	29 (34.5)	**51**	**33**	0.640
			19 (37.3)	10 (30.3)	
**Body mass index (kg/m²), mean (SD)**	**80**	25.6 (6.3)	**49**	**31**	0.852
			25.7 (4.8)	25.4 (8.1)	
**Hemophilia severity, n (%)**	**85**		**51**	**34**	
Moderate		14 (16.5)	6 (11.8)	8 (23.5)	0.232
Severe		71 (83.5)	45 (88.2)	26 (76.5)	
**Time since diagnosis of hemophilia (months), mean (SD)**	**53**	342.7 (168.6)	**36**	**17**	0.453
			354.8 (174.0)	317.1 (158.5)	
**Age at diagnosis of hemophilia (years), mean (SD)**	**53**	3.5 (8.5)	**36**	**17**	0.230
			4.5 (10.1)	1.5 (2.1)	

Characteristic	N		Follow-up completed (12 months)		p-value[a]
			Yes	No	
**Treatment (prophylaxis) for hemophilia, n (%)**	**85**	77 (90.6)	**51**	**34**	0.055
			49 (96.1)	28 (82.4)	
**Comorbidities, n (%)**	**85**		**51**	**34**	
Any		41 (48.2)	31 (60.8)	10 (29.4)	**0.007**
Hemophilic arthropathy		14 (16.5)	12 (23.5)	2 (5.9)	**0.038***
Hepatitis C		10 (11.8)	9 (17.6)	1 (2.9)	**0.045***
HIV		8 (9.4)	6 (11.8)	2 (5.9)	0.467
Hypertension		8 (9.4)	7 (13.7)	1 (2.9)	0.137
Obesity		4 (4.7)	2 (3.9)	2 (5.9)	>0.999
Hypercholesterolemia		4 (4.7)	3 (5.9)	1 (2.9)	0.647
Diabetes		4 (4.7)	3 (5.9)	1 (2.9)	0.647
**Social assistance for hemophilia n (%)**	**84**	6 (7.1)	**51**	**33**	0.396
			5 (9.8)	1 (3.0)	
**Caregiver (yes), n (%)**	**85**	12 (14.1)	**51**	**34**	>0.999
			7 (13.7)	5 (14.7)	

SD, Standard deviation; HIV, juman immunodeficiency virus

[a] Wilcoxon signed-rank test

*All p-values < 0.05 marked with an asterisk should be interpreted with caution, as the sample sizes for some categories are less than 3 observations.

Table S3. Caregivers' Evaluation of Quality of Life, Work Productivity, and Anxiety/Depression

Humanistic outcomes	Baseline		Month 12	
	N	Mean (SD)	N	Mean (SD)
**HRQoL[a]**	12	66.9 (29.1)	7	71.1 (25.2)
**Work productivity[b]**				
% Work time missed	6	1.9 (4.5)	3	33.3 (57.7)
% Impairment while working	6	3.3 (5.2)	3	0 (0)
% Overall work impairment	6	5.2 (5.7)	3	33.3 (57.7)
% Activity impairment	12	20.8 (22.7)	7	35.7 (34.1)
**Anxiety and depression[c]**				
Anxiety subscale	12	8.0 (5.4)	7	10.0 (5.9)
Depression subscale	12	5.8 (4.0)	7	6.6 (5.7)

[a]EuroQol-5D-5L (EQ-5D-5L) Questionnaire

[b]Work Productivity and Activity Impairment - General Health (WPAI-GH) Questionnaire

[c]Hospital Anxiety and Depression Scale (HADS)

SD, Standard deviation; %, percentage
